# Health Tract, No. 18

**Published:** 1862-01

**Authors:** 


### HEALTH TRACT, No. 18.

### THE BEST HAIR-WASH.

(*From Hall's Journal of Health*, 42 *Irving Place*, *New-York.*)

A Southern correspondent says: "In the matter of a hair-wash, in a recent number of the Journal of Health, I have received a thousand times its cost, and it has also been a benefit to many others."

Make half a pint soap-suds with pure white soap and warm water, on rising any morning; but before applying it, brush the whole scalp well, while the hair is perfectly dry, with the very best Russia bristle brush, scrub back and forth with a will, let not any portion of the surface escape. When brushing the top and front, lean forward, that the particles may fall. After this operation is finished, strike the ends of the bristles on the hearth or on a board, next pass the coarse part of the comb through the bristles; next, brush or flap the hair back and forth with the hand until no dust is seen to fall; then with the balls of the fingers dipped in the soap-suds, rub the fluid into the scalp and about the roots of the hair; do this patiently and thoroughly. Finally, rinse with clear water, and absorb as much of the water from the hair as possible with a dry cloth; then (after allowing the hair to dry a little more by evaporation, but not to dry entirely) dress it as usual, always, under all circumstances, passing the comb through the hair slowly and gently, so as not to break any one off, or tear out any one by the roots.

By this operation the alkali of the soap unites with the natural oil of the hair, and leaves it perfectly clean and beautifully silken, and with cold water washings of the whole head and neck and ears every morning, it will soon be found that the hair will "dress" as handsomely as if "oiled to perfection;" with the great advantage of conscious cleanliness, giving, too, the general appearance of a greater profusion of hair than when it is plastered flat on the scalp, with variously scented hog's fat, as is the common custom.

It has been recently established, in a court of justice in the city of New-York, that one of the most popular hair-washes ever known was made by adding a little alcohol, scented with a perfume, to common soap-suds.

**A better hair-wash is a tea-spoonful of powdered borax in half a glass of warm water, applied as above weekly, with a good use of the hair-brush daily, using a comb only to straighten out the hair, touching the scalp but very lightly indeed, (otherwise it makes it rough and injures the roots of the hair.) This will prevent DANDRUFF, which answers to what is deposited from the curry-comb of a horse, being the dead scales, dried oil, perspiration, and dust of the scalp. Nothing can ever make hair grow on a shining scalp. Pure soft water will make the hair "dress" better than any thing else in nature. It is an unclean scalp that "rots" the hair and imparts an unpleasant odor. If good health, a clean scalp, and cutting off the extreme ends of the hair monthly does not make it grow, nothing else can.**

